# Effect of Degassing Parameters on Mechanical Properties of EN AC-46000 Gravity Die Castings

**DOI:** 10.3390/ma15238323

**Published:** 2022-11-23

**Authors:** Grzegorz Gumienny, Bogusław Pisarek, Tomasz Szymczak, Jakub Gawroński, Paweł Just, Ryszard Władysiak, Cezary Rapiejko, Tadeusz Pacyniak

**Affiliations:** Department of Materials Engineering and Production Systems, Lodz University of Technology, 90-924 Lodz, Poland

**Keywords:** degassing, Al-Si alloys, mechanical properties, hydrogen content

## Abstract

The article is devoted to the optimization of the degassing parameters of the AlSi9Cu3(Fe) alloy. The alloy was degassed with a solid degasser (Ecosal) and nitrogen or argon. The variable parameters were time and type of degasser. The test castings were made in permanent molds with an internal diameter of 25 mm and a length of 150 mm. The effect of the degassing time and the amount of degasser on the mechanical properties, as well as the hydrogen content and density index were investigated. The ALU SPEED TESTER developed by FMA was used to test the hydrogen content and the density index. Magmasoft software was used to design the geometry of the test castings. A significant effect of the solid degasser and degassing time on both the density index and the hydrogen content was demonstrated. Replacing nitrogen with argon did not bring any significant improvement in the above-mentioned parameters. The effect of degassing parameters on the mechanical properties of the EN AC-46000 alloy was much less significant, but was still visible. The optimal degassing parameters needed to obtain the highest strength parameters of the EN AC-46000 gravity die castings were determined.

## 1. Introduction

Degassing of aluminum alloys is one of the basic stages of their correct preparation before pouring them into the mold. Its purpose is to remove hydrogen, oxides, borides, nitrides, and spinels from the alloy as they significantly reduce the mechanical properties of Al alloys. Be and Ca can also show a harmful effect, causing porosity of the castings. In phosphorus-modified alloys, sodium and calcium increase the casting shrinkage [1]. Mainly inert gases and chemical compounds are used in the degassing process, but also ultrasound, infrasound, and magnetic field, as well as low and high pressure [2,3,4,5,6,7,8,9,10,11,12,13,14,15,16]. The following inert gases are used: nitrogen, argon, chlorine, and, optionally, their mixtures. Degassing with nitrogen requires a relatively long time to clean the liquid alloy. Argon is more effective but more expensive, thus its use is sometimes limited in industry. Chlorine is very harmful to both the environment and to humans; therefore, it is usually used as an additive to nitrogen or argon [1]. Rotary degassing is currently one of the most widely used alloy treatment techniques in the foundry industry [17]. The degassing process is also of great importance for the removal of oxide layers (so-called bifilms), which are largely responsible for the metallurgical quality of the liquid alloy [18,19,20,21,22]. In [23], it was shown that argon exerts the most favorable influence on the alloy’s quality in this respect.

As a rule, it is assumed that time is the most important parameter in degassing. On the one hand, it must ensure the removal of harmful gases from the liquid metal, and on the other hand, it must prevent re-gassing. Sometimes, specific methods are used for the degassing process, such as high shear melt conditioning (HSMC) technology where a simple rotor–stator mixer is used [24,25,26,27]. The work of [24] shows the advantages of this method in the degassing of an Al-Si alloy in comparison with the classic degassing with the use of a rotor. Information on the effect of hydrogen on the mechanical properties of aluminum alloys can be found in the literature. Some of them, however, concern alloys subjected to the hydrogen cathodic charging process [28,29]. The work of [25] showed a significant effect for the type of degassing on the tensile strength (the highest values were obtained with degassing using the HSMC method); at the same time, the degassing method had no effect on the yield point. As time passed after the degassing process, a decrease in tensile strength, yield point, and elongation was observed. The fracture surfaces of the post-mortem tensile bars showed both blowholes and oxide layers, responsible for lowering the strength properties of the tested alloy. In addition to the above-mentioned tests, aluminum casting alloys were the subject of many other tests in terms of their degassing and hydrogen content [30,31,32,33].

Although the degassing process seems to be well known, the selection of the right parameters is usually the domain of foundry practice. The problem may be the correct selection of the above-mentioned parameters when using two types of degassers. These parameters are usually selected experimentally in the given production conditions of the foundry. Foundries often carry out the degassing process in a conventional manner, as they are not always able to apply modern, often expensive, solutions. Such a problem arose at the company Wifama-Prexer, Łódź, Poland, and it was resolved as part of the project. Therefore, the aim of this study is to investigate the effect of the amount of solid degasser and the time of degassing with nitrogen or argon on the mechanical properties of the EN AC-46000 alloy. Additionally, the effect of degassing parameters on the density index and hydrogen content in the tested alloy was investigated.

## 2. Materials and Methods

The EN AC-46000 alloy was used for the tests. It is a typical hypoeutectic Al-Si alloy for high pressure die casting (HPDC) with the designation AlSi9Cu3(Fe), according to EN 1706. The experiments were carried out using the Box–Wilson optimization method (two-level planning). At the stage of the preliminary tests, optimization of the chemical composition was carried out, narrowing the range of variability of Si, Cu, and Mg (in relation to the composition specified in the EN 1706 standard), in order to maximize the mechanical properties of the tested alloy.

For the degassing process of the investigated alloy with an optimal chemical composition, the extreme object outputs were searched for the alloy quality factors:

- DI density index (minimum),
- Mechanical properties (maximum tensile strength R_m_, yield strength R_p0.2_, elongation A, and hardness HBW).

During optimization, a non-dominant solution was sought, i.e., improvement, which is not possible because of any of the applicable criteria without the deterioration of any of the other criteria. In the search for a solution, the variable normalization method was used to evaluate a finite number of variants to choose from, namely the zero unitarization method. The optimization process was carried out in the following stages:

- Analysis of output data and selection of diagnostic variables to evaluate the parameters of the compared solutions;
- Normalization of diagnostic variables;
- Aggregation of several normalized diagnostic variables to one assessment (by summation);
- Searching for the optimal solution for the aggregate value of the objective function.

In this method, for the purpose of the normalization and aggregation of the objective function, all of the variables used in the evaluation of individual criteria were divided into three classes:

- Stimulants (variables, the increase of which should be associated with an increase, and a decrease with a decrease in the assessment of the phenomenon);
- Destimulants (variables, the increase of which should be associated with a decrease, and a decrease with an increase in the assessment of the phenomenon);
- Nominants (no variables of this class were identified during the research).

The zero unitarization method is characterized by adopting a fixed reference point, which is in the range of a given normalized variable. The range R for each feature “j” is as follows:

(1) $$R(X_{j})=\max\left(x_{\mathrm{ij}}\right)-\min\left(x_{\mathrm{ij}}\right)$$

for all “i” items. Because of this approach, the range of the standardized criterion’s feature is constant and is equal to 1.

The criteria normalization process (i.e., their conversion to comparable values for all criteria) was carried out for the following:

- stimulants—the variable is normalized according to the following formula:

(2) $$z_{\mathrm{ij}}=\frac{x_{\mathrm{ij}}-\min\left(x_{\mathrm{ij}}\right)}{\max\left(x_{\mathrm{ij}}\right)-\min\left(x_{\mathrm{ij}}\right)}=\frac{x_{\mathrm{ij}}-\min\left(x_{\mathrm{ij}}\right)}{R(X_{j})}$$

- destimulants—the variable is normalized according to the following formula:

(3) $$z_{\mathrm{ij}}=\frac{\max\left(x_{\mathrm{ij}}\right)-x_{\mathrm{ij}}}{\max\left(x_{\mathrm{ij}}\right)-\min\left(x_{\mathrm{ij}}\right)}=\frac{\max\left(x_{\mathrm{ij}}\right)-x_{\mathrm{ij}}}{R(X_{j})}$$

The final value of the aggregated objective function, OF, according to which the optimal solution is sought, is as follows:

(4) $${\mathrm{FC}}_{j}=\sum \left(z_{\mathrm{ij}}\cdot w_{j}\right)\to \max$$

for each “j” criterion and “i” assessed items, where w_j_ is the weight of the “j” criterion.

The following criteria were adopted in multi-criteria optimization:

1. Maximization:
  - Modulus of longitudinal elasticity E, GPa;
  - Proof stress R_p0.2_, MPa;
  - Tensile strength R_m_, MPa;
  - Elongation A, %.
2. Minimalization:
  - DI density index, %.
3. Weight factors:
  - No properties were distinguished (w_j_ = 1).

Preliminary studies have shown that Si, Cu, and Mg exerted the greatest effect among the alloy’s constituent elements on its mechanical properties. It has also been shown that in order to obtain the best strength properties, the chemical composition of the alloy should be narrowed down to 9.0 ÷ 10.0% Si; 2.0 ÷ 2.5% Cu, and 0.3 ÷ 0.4% Mg. Therefore, the tests assumed the above-mentioned concentrations of Si, Cu, and Mg, while the content of the other elements corresponded to the EN 1706 standard. Two types of degassers were used: solid, Ecosal Al 113S, and gas, argon or nitrogen. Ecosal Al 113S is a NaCl + KCl-based degasser for the purification and deslagging of aluminum alloys. It is a mixture based on alkaline chlorides and alkaline earth chlorides with the addition of alkali halides and alkaline earth halides and other salts. Its composition is as follows: sodium chloride/potassium chloride-base, sodium and potassium sulphate or nitrate, other halides such as difluorides, fluorspar, as well as carbonates. The exact chemical composition of Ecosal Al 113S is a trade secret of the manufacturer. The maximum amount of solid degasser was assumed to be 1% of the weight of the treated metal (with a step of 0.5%), while the maximum degassing time with nitrogen or argon was set at 15 min (with a step of 7.5 min).

The chemical composition of the tested alloy is presented in Table 1.

**Figure 1:**
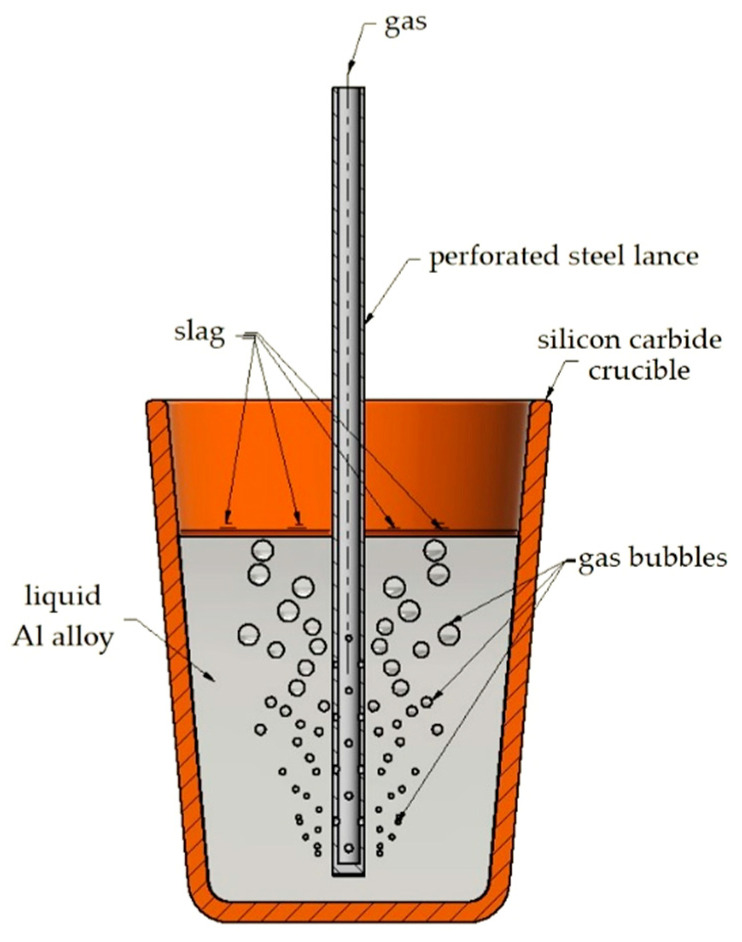
Scheme of the gas degassing process with the use of a perforated steel lance.

The charge was melted in an Elkon PI 30 (Elkon, Poland) induction furnace with a cemented carbide crucible with a capacity of 7 kg of Al alloy. The charge was ingots of the EN AC-46000 alloy. After smelting, the metal was superheated to 750 ± 10 °C. In order to obtain the assumed content of Si, Cu, and Mg, Si or Al and Cu were added into the liquid alloy as technically pure elements, while magnesium was added as the AZ91 alloy. The degassing process was carried out in a furnace crucible with a solid degasser Ecosal Al113S and/or a gas degasser, i.e., nitrogen or argon. The gas was blown into the liquid alloy with a perforated steel lance, as shown in Figure 1. The gas pressure flowing into the steel lance was constant.

**Figure 2:**
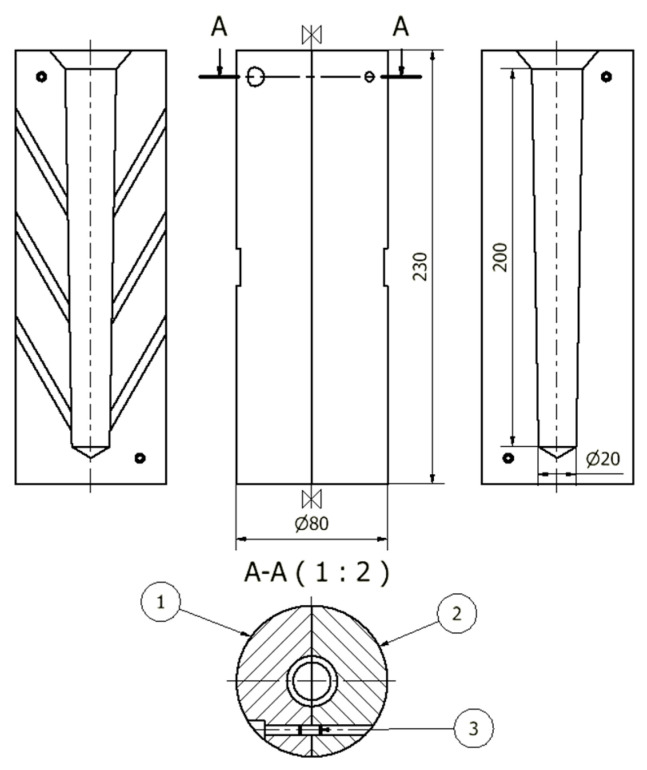
The shape and dimensions of the permanent mold for test castings ϕ20: 1—left part, 2—right part, 3—locating pin.

After degassing, the temperature of the liquid metal was lowered to 700 ± 10 °C and the steel permanent mold was poured. The mold cavity was cylindrical in shape with a diameter of 25 mm and a length of 150 mm. Three permanent molds heated to a temperature of 180 °C were used for the mechanical properties tests. Figure 2 shows the shape and dimensions of the permanent mold.

Both the lance and the permanent molds (three pieces) were each time covered with a mixture of TiO_2_ + Na_2_O · NO_2_Si/titanium dioxide + sodium water glass/(titanium oxide melting point 1850 °C) by compressed air spraying, and then dried before contact with the liquid alloy.

**Figure 3:**
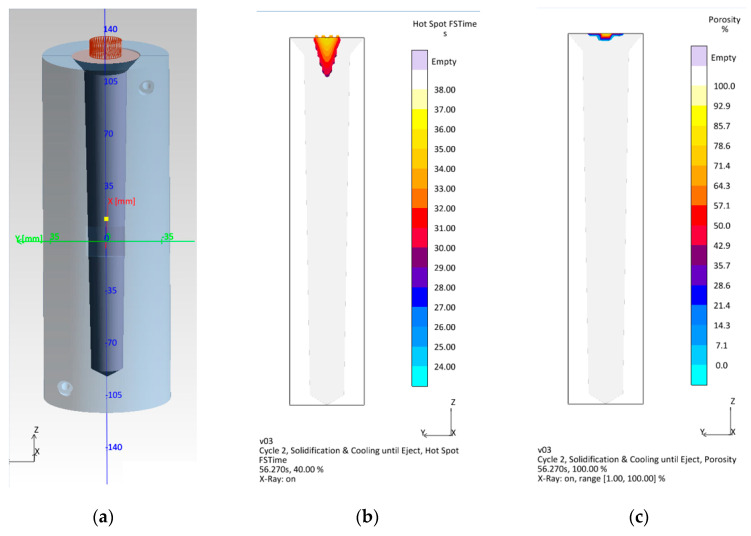
Simulation of the test casting solidification in the permanent mold: (**a**) model of the permanent mold casting system, (**b**) solidification time (FSTime) of the alloy in the hot spot, (**c**) porosity of the casting.

The geometry of the test castings (permanent molds cavities), from which the specimens for testing the strength properties were made by machining, were selected on the basis of the simulation results in MAGMASOFT^®^ (MAGMA Gießereitechnologie GmbH, Aachen, Germany) of the EN AC-46000 alloy solidification (version number 5.5.0.2). For the purposes of the simulation, the initial temperature of the cast alloy was set at 670 °C, while the initial temperature of the permanent mold was set at 180 °C. The control condition with regard to the dimensions of the permanent mold cavity was the lack of shrinkage porosity and hot spots in the areas forming the measuring section of the strength specimen. The pouring time was set to 3 s. The temperature of the casting when the mold was opened was set at 450 °C, which guaranteed the sufficient strength of the casting during its removal from the mold. The delay time was set to 15 s. This is the time needed to pour the ladle with the liquid melt from the furnace. The simulation results in MAGMASOFT^®^ are shown in Figure 3.

Figure 3 shows that with the assumed shape of the permanent mold cavity, the shrinkage porosity appears only in the ingate area (Figure 3c), which does not affect the quality of the castings. Figure 3b shows that the hot spots are located only in the sprue. The above-described preliminary tests made it possible to be sure that the casting defects arising during the production of test castings would come from gases dissolved in the alloy as well as from oxide layers formed as a result of the contact of the liquid alloy with air.

**Figure 4:**
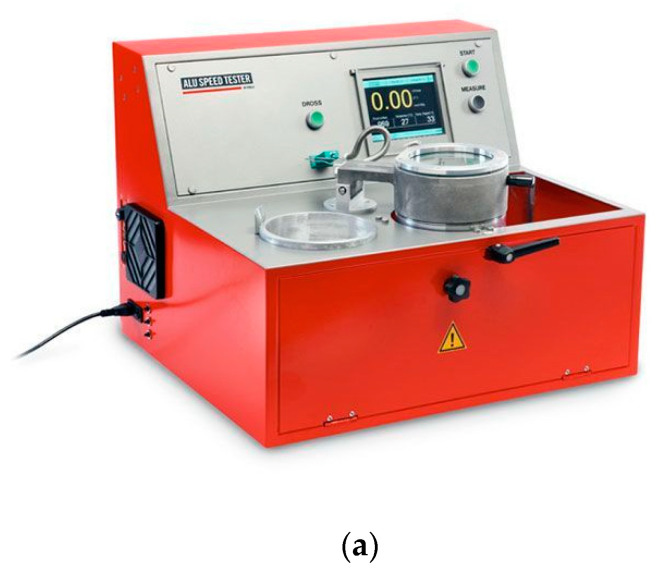

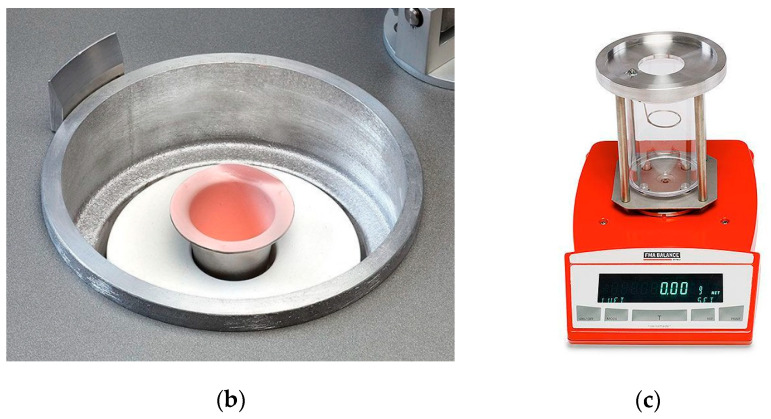
Alloy quality test stand: (**a**) Alu Speed Tester, (**b**) metal mold for testing alloy density, and (**c**) FMA Balance.

Density index and hydrogen content tests were performed with the use of ALU SPEED TESTER (FMA Mechatronic Solutions AG, Liechtenstein); it is shown in Figure 4.

The density index DI was determined from the following formula:

(5) $$\mathrm{DI}=\frac{D_{\mathrm{atm}}-D_{80\mathrm{mbar}}}{D_{\mathrm{atm}}}\cdot 100\%$$

where:

D_atm_—density of sample solidified under atmosphere, g/cm^3^;
D_80mbar_—density of the sample solidified under a vacuum (80 mbar), g/cm^3^.

Because of the long measurement time, the results of the density index and hydrogen content are values from the individual measurements.

Metallographic tests were performed on specimens cut out from the gravity die castings. The microsections were etched with a 2% aqueous solution of hydrofluoric acid (HF). The microstructure was examined using an Eclipse MA200 optical microscope (Nikon, Tokyo, Japan) with ×1000 magnification.

**Figure 5:**
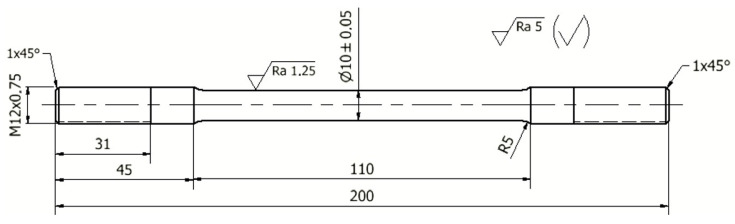
Dimensions of the specimen for the strength tests.

The mechanical properties were made in accordance with ISO 6892-1: 2016-09 on an INSTRON 4485 (Instron, Norwood, MA, USA) testing machine on specimens with a diameter of 10 mm and a length of 110 mm. The dimensions of the specimen for testing the strength of the properties are shown in Figure 5.

Alloy hardness was measured by Brinell method using an HPO-2400 hardness tester (WPM LEIPZIG, Leipzig, Germany), according to EN ISO 6506-1:2014-12. The diameter of the ball was 2.5 mm, the load was 613 N, and the static load holding time was 30 s. Hardness tests were performed on a separate specimen cut out for this purpose from a test casting. The values of the mechanical properties presented in the test results are the arithmetic mean of three measurements.

## 3. Results and Discussion

**Figure 6:**
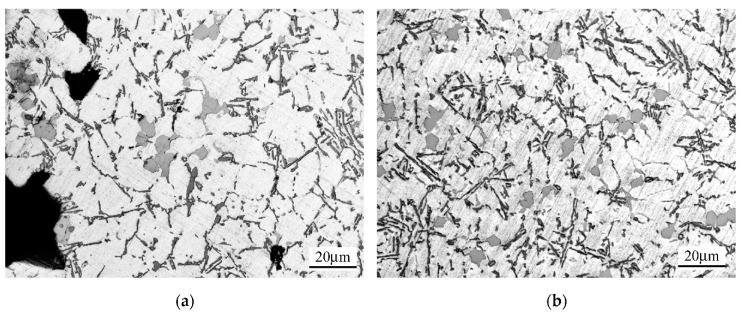
The microstructure of the EN AC-46000 alloy in a permanent mold casting: non-degassed (**a**) and degassed with Ecosal (1%) and nitrogen for 15 min (**b**).

Figure 6 shows the microstructure of the EN AC-46000 alloy non-degassed (a) and degassed with Ecosal (1%) and nitrogen for 15 min (b) from the permanent mold.

Figure 6a,b shows that the microstructure is typical for a hypoeutectic alloy with clearly visible dendrites of the primary α phase and the eutectic mixture (α + β). Apart from the above-mentioned phases, the Al_2_Cu phase is visible, among others. The dark areas visible in Figure 6a are discontinuities in the microstructure caused mainly by the presence of hydrogen in the alloy. They come only from the presence of gas not removed in the degassing process—the shape of the test casting, as mentioned in Section 2 (Materials and Methods), excluded the occurrence of casting defects caused by alloy shrinkage.

**Figure 7:**
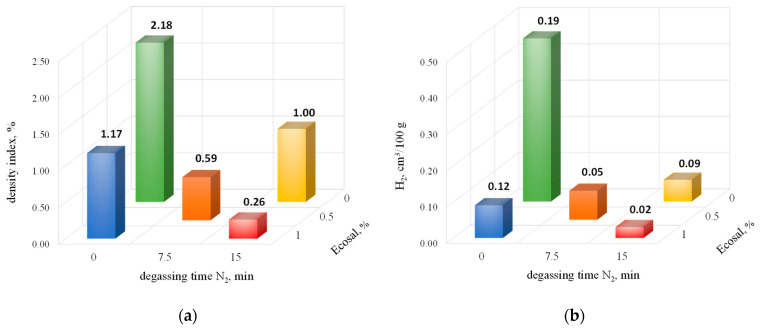
The density index (**a**) and hydrogen content (**b**) vs. degassing parameters (with Ecosal and N_2_).

In Figure 7, the density index as well as hydrogen content vs. degassing parameters (with Ecosal and N_2_) are presented.

The data presented in Figure 7a show a very significant effect of both the time of degassing with nitrogen and the amount of solid refiner on the density index. It should be noted that the impact of both of the above-mentioned factors is similar. With the degassing parameters tested, the maximum reduction in the density index was achieved by as much as about 90%.

Figure 7b also shows a significant effect of the degassing parameters on the hydrogen content in the alloy. With a nitrogen degassing time of 15 min and the maximum amount of solid refiner, a value of 0.02 cm^3^/100 g was obtained, which means a reduction in the hydrogen content by almost 90%.

**Figure 8:**
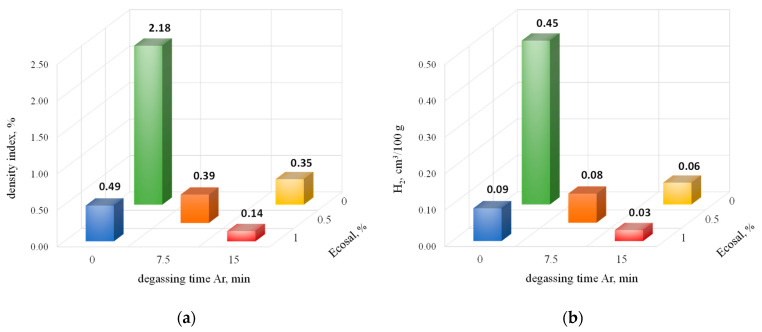
The density index (**a**) and hydrogen content (**b**) vs. degassing parameters (with Ecosal and Ar).

In Figure 8, the density index as well as hydrogen content vs. degassing parameters (with Ecosal and Ar) are presented.

The data in Figure 8 show that the change of the degasser from nitrogen to argon brought some improvement in terms of the density index, which at the maximum time of degassing and the highest amount of solid degasser decreased by more than 50%. When testing the hydrogen content in the alloy, the results were similar to those for degassing with nitrogen.

**Figure 9:**
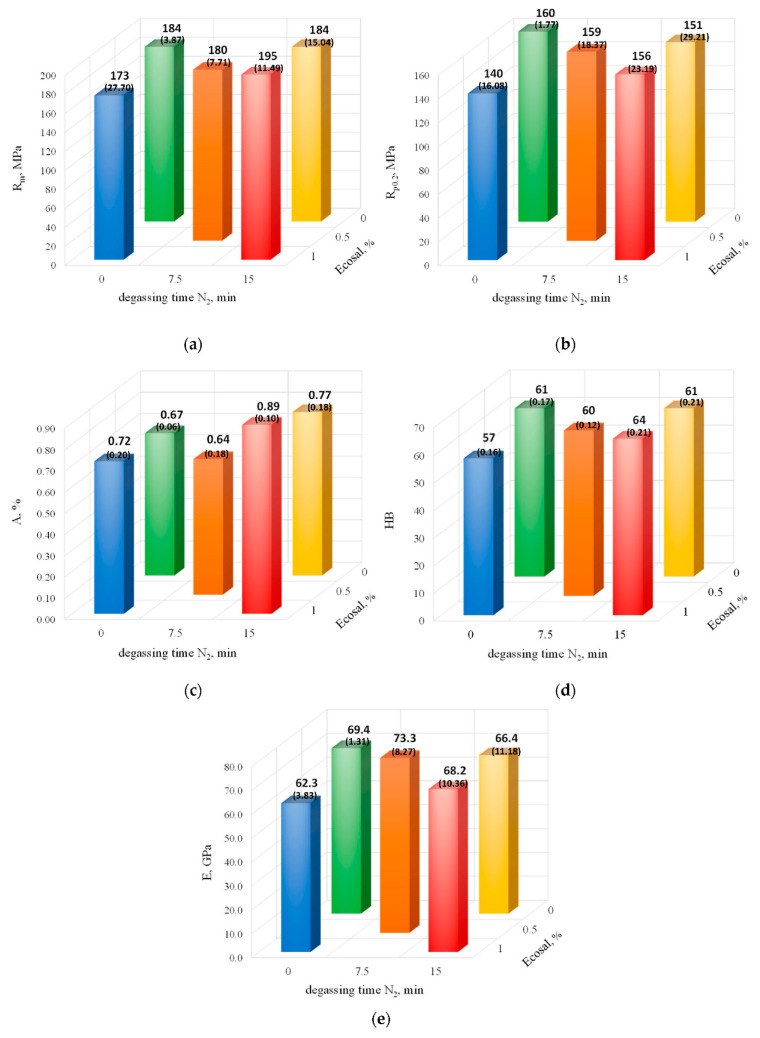
The tensile strength (**a**), yield point (**b**), elongation (**c**), Brinell hardness (**d**), and Young’s modulus (**e**) vs. degassing parameters (with Ecosal and N_2_). The standard deviation values are given in brackets.

Figure 9 shows the effect of the degassing parameters (with Ecosal and N_2_) on the mechanical properties of the EN AC-46000 alloy.

The data presented in Figure 9a–e show some effect of the degassing parameters on the mechanical properties; however, it is not as clear as in the case of the hydrogen content or the density index. In the case of degassing only with Ecosal, even a reduction in mechanical properties was observed compared with the non-degassed alloy, which should be explained by its lack of effectiveness under the tested conditions. It is visible, for example, in the case of the tensile strength and yield point (Figure 9a,b). When degassing only with nitrogen, this effect was much less visible—a slight decrease in properties occurred only in the case of R_p0.2_ and E. The joint application of Ecosal and nitrogen has already brought benefits, but not for all of the properties tested. An increase in tensile strength (about 6%), elongation (relatively by about 33%), and hardness (about 5%) was observed. In the remaining two cases, a slight decrease in properties was observed. The research shows that there is no direct correlation between the density index, hydrogen content, and mechanical properties.

**Figure 10:**
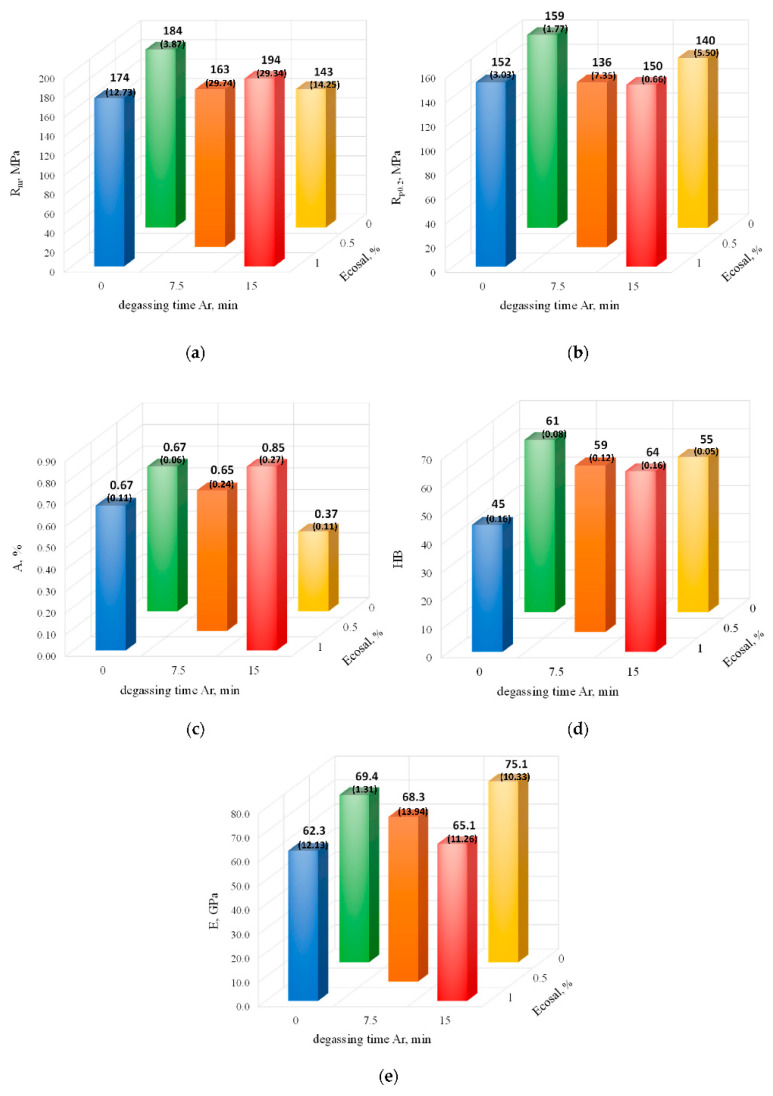
The tensile strength (**a**), yield point (**b**), elongation (**c**) Brinell hardness (**d**), and modulus of elasticity (**e**) vs. degassing parameters (with Ecosal and Ar). The standard deviation values are given in brackets.

Figure 10 shows the effect of the degassing parameters (with Ecosal and Ar) on the mechanical properties of the EN AC-46000 alloy.

A comparison of the data in Figure 9 and Figure 10 shows that replacing nitrogen with argon did not bring the expected benefits. The properties tested were either similar or even lower compared with the nitrogen-degassed alloy. As in the case of the nitrogen-degassed alloy, there was an increase in tensile strength (about 5%), elongation (relatively by about 27%), and hardness (about 5%). The research shows that (as in the case of degassing with nitrogen) there was no direct correlation between the density index, hydrogen content, and mechanical properties of the tested alloy.

**Figure 11:**
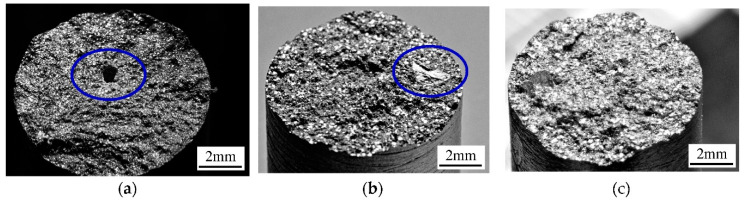
The fractures of tensile specimens: undegassed (**a**), after degassing with Ecosal (0.5%) and N_2_ in 7.5 min, (**b**), after degassing with Ecosal (1%) and N_2_ in 15 min. (**c**) The blue ellipse shows the blowhole (**a**) and the oxide layer (**b**).

Because of the relatively small changes in the strength properties before and after the degassing process, the fractures of the tensile specimens were also examined. Therefore, Figure 11 shows the fractures of the selected specimens after the tensile test.

The data presented in Figure 11 show that in the non-degassed specimens, there were blowholes (marked with an ellipse in Figure 11a), which reduced the effective cross-section of the strength specimens. Figure 11b shows the oxide layer (also marked with an blue ellipse), which remained despite the alloy degassing process. These layers are formed very quickly on the surface of the liquid metal/air interface, also during metal transfer (both during pouring from the furnace into the ladle as well as during pouring the mold), and had a significant impact on the mechanical properties of the alloy. The movement of the liquid metal (both during pouring and convection after pouring the mold) broke the oxide layers; some of them remained trapped inside the casting, which reduced its mechanical properties. Despite the lack of visible defects in Figure 11c, on the basis of the mechanical property tests, it can be assumed that oxide layers can occur in the degassed alloy within 15 min and after using Ecosal in the maximum amount tested.

Summing up, it should be stated that the degassing process had a decisive influence on the hydrogen content in the alloy (which is obvious); however, its effect on the mechanical properties of gravity die castings was much smaller. The analysis of the test results showed that the assumed degassing time range did not result in the alloy re-gassing, despite the fact that it was relatively long. It should be noted that the use of two degreasers, both solid and gas, should have a positive effect on the mechanical properties of aluminum alloys from a permanent mold. This facilitates not only the removal of gases dissolved in the melt, but also the removal of oxide films (bi-films) which are very harmful in terms of their mechanical properties.

## 4. Conclusions

From the data presented in this paper, the following conclusions emerge:

1. Degassing with a solid degasser in the amount of 1% of the alloy and nitrogen within 15 min reduced the hydrogen content by almost 90%; replacing nitrogen with argon increased this value to about 93%.
2. Degassing with both a solid and gas degasser may have increased the mechanical properties of the EN AC-46000 alloy; for tensile strength, this increase did not exceed 6%; this relatively small increase could be explained by the remaining oxide layers inside the casting.
3. To intensify the degassing process, it is strongly recommended to use two types of degasser: solid and gas; this is important in terms of increasing the mechanical properties.
4. The use of two degassers, both solid and gas, may not result in complete removal of the oxide layers, which significantly reduced the strength properties of the aluminum alloy.

## Figures and Tables

**Table 1:** Chemical composition of the EN AC-46000 alloy.

Chemical Composition, wt.%										
Si	Cu	Zn	Fe	Mg	Mn	Ni	Ti	Pb	Sn	Cr
9.4 ÷ 9.7	2.2 ÷ 2.4	0.9 ÷ 1.2	0.91 ÷ 0.97	0.29 ÷ 0.37	0.22 ÷ 0.23	0.11 ÷ 0.12	0.042 ÷ 0.050	0.067 ÷ 0.071	0.019 ÷ 0.023	0.030 ÷ 0.032

## Data Availability

Not applicable.
